# Miscellany

**Published:** 1892-03

**Authors:** 


### Miscellany.

---

MR. HOWELLS' NEW WORK.—The announcement that Mr. Howells will leave *Harper's Magazine*, to take editorial charge of the *Cosmopolitan*, on March 1st, calls attention to the process of building up the staff of a great magazine. Probably in no monthly has the evolution been so distinctly under the eyes of the public as in the case of the *Cosmopolitan*. The first step after its editorial control was assumed by Mr. John Brisben Walker, was to add to it Edward Everett Hale, who took charge of a department called "Social Problems," subjects concerning which the greatest number of people are thinking today. Mr. Hale, who is a student, a fair-minded man, a thorough American, and a man of broad sympathies, has filled this position in a way to attract the attention not only of this country, but of leading European journals. Some months later, a department was established called "The Review of Current Events." To take charge of this, a man was needed who should be familiar not only with the great events of the past thirty years, but who knew personally the leading men of both the United States and Europe who could interpret motives and policies. Murat Halstead accepted this position with the distinct understanding that his monthly review should be philosophical and never partisan. The next step in the history of the *Cosmopolitan* was the placing of the review of the intellectual movement of the month in the hands of Mr. Brander Matthews, who for some time has been recognized as one of the two or three ablest critics in the United States.

Finally came the acceptance of the editorship conjointly with Mr. Walker, by Mr. William Dean Howells. Mr. Howells, who is recognized universally as the foremost American of letters, upon the expiration of this contract with Harper Brothers, on the first of March, will take in hand the destinies of a magazine which promises to exercise a share of influence with the reading classes of the United States. His entire services will be given to the *Cosmopolitan*, and everything he writes will appear in that magazine during the continuance of his editorship.

W. B. SAUNDERS, publisher, of Philadelphia, announces the two following important works as being in the press :

"An American Text-book of Surgery," by Professors Keen, White, Burnett, Conner, Dennis, Park, Nancrede, Pilcher, Senn, Shepherd, Stimson, Thomson, and Warren. Forming one handsome royal octavo volume of about 1200 pages (10x7 inches), profusely illustrated with wood-cuts in text, and chromo-lithographic plates ; many of them engraved from original photographs and drawings furnished by the authors. Price, cloth, $7.00 ; sheep, $8.00.

"An American Text-book of the Theory and Practice of Medicine according to American Teachers," edited by Wm. Pepper, M. D., LL. D., Provost of the University of Pennsylvania. To be completed in two handsome royal octavo volumes of about 1000 pages each, with illustrations to elucidate the text wherever necessary. Price, per volume, cloth, $5.00 ; sheep, $6.00 ; half Russia, $7.00. For sale by subscription only.

---

EARACHE.—Take five parts of camphorated chloral, thirty parts of glycerine, and ten parts of oil of sweet almonds. A piece of cotton is saturated and introduced well into the ear, and it is also rubbed behind the ear. The pain is relieved as if by magic, and if there is inflammation it often subsides quickly.—*Medical Brief.*

---

THE N. Y. PHYSICIANS' MUTUAL AID ASSOCIATION now has one thousand members, composed only of regular physicians in good standing.

It assists needy members, aids widows of deceased members. Will pay $1,000 at death of a member as soon as the membership reaches 1,100.

Initiation fee is from $2.00 to $5.00, according to age, and no candidates are received over fifty years of age.

Opportunity is offered to join the association at any time by applying to Dr. W. G. Gregory or Dr. S. A. Dunham.

